# Improving biosafety measures in high containment laboratories and patient care: a systematic analysis of *Orthoebolavirus* and *Henipavirus* stability

**DOI:** 10.3389/fpubh.2025.1648115

**Published:** 2025-11-03

**Authors:** Denise-Carina Kranz, Joo-Hee Wälzlein, Katharina Kimmerl, Andreas Kurth, Susann Kummer

**Affiliations:** Center for Biological Threats and Special Pathogens, Robert Koch-Institute, Berlin, Germany

**Keywords:** stability, infectious particles, Ebola virus, Nipah virus, biosafety

## Abstract

Emerging and re-emerging high-risk pathogens demand strong biosafety protocols for both patient care and laboratory practices. This study aimed to produce experimental data to support evidence-based guidelines for improving safety measures related to *Orthoebolavirus* and *Henipavirus*. Viruses in solution were applied to materials commonly found in hospital and lab settings—stainless steel, glass, plastics, cotton, nitrile and rubber gloves, and protective suits. Stability and infectivity were monitored over time under two conditions: (1) a typical indoor lab/hospital environment and (2) warmer, humid conditions resembling a European summer. While laboratory and clinical environments are typically climate-controlled, inclusion of the higher temperature and humidity condition provides comparative data relevant for situations where environmental controls may be less consistent, such as in public or outdoor settings. Results show that virus stability depends on both the suspension medium and the surface texture. Personal protective materials generally retained the virus for shorter durations. No viable virus was found after 112 days, with most becoming undetectable by day 28. Routine chemical disinfection protocols remain the primary biosafety measures, and our findings offer key insights for refining disinfection strategies and enhancing biosafety in high-containment settings and clinical care environments.

## Introduction

With a growing global population and the accelerating effects of urbanization and globalization, the risk of emerging infectious diseases continues to rise. Among these, viruses classified as Risk Group 4 (RG-4)—such as members of the *Orthoebolavirus* and *Henipavirus* genera—pose particular concern due to their high pathogenicity and potential for epidemic spread (1–5). Current recommendations and guidelines for disinfection procedures in clinical and laboratory environments are largely based on standardized protocols developed from systematic testing of chemical inactivation methods. However, real-world contamination events are highly variable and may not always reflect the ideal conditions under which such disinfection protocols were validated. Studying the environmental stability of viruses—specifically how temperature and humidity affect the persistence (tenacity) of viral particles on various surfaces—is essential for supporting outbreak control strategies. Such investigations also help inform appropriate adjustments in disinfectant application, particularly in cases of spills with infectious materials (1, 6). In this context, previous studies have explored selected aspects of the environmental stability and recovery of infectious *Orthoebolavirus* and *Henipavirus* particles on contaminated surfaces (7–13). Nonetheless, systematic generated data under well-defined and reproducible laboratory conditions remain scarce, especially for highly pathogenic agents (6, 14–16). Furthermore, existing data show considerable variability, likely influenced by numerous factors, including the origin of the viral material [e.g., patient-derived vs. cultured virus in liquid media (17, 18)], the surface materials tested, and the experimental design itself. These discrepancies limit the comparability of results across studies and limit the applicability to standard biosafety or infection control protocols. In the present study, we aim to address this gap by systematically assessing the surface stability and retention of *Orthoebolavirus* and *Henipavirus* particles under two controlled climate conditions. The tested surfaces were selected to reflect materials commonly found in medical and laboratory settings. Our goal is to provide robust, reproducible data that may serve as a scientific basis for context-specific risk assessments and support biosafety considerations—particularly in scenarios involving contaminated biological material deposited onto surfaces.

## Materials and methods

### Virus strains

Viruses used in this study were Ebola virus-GFP, EBOV-GFP, (3.86 × 10^6^ PFU/mL) provided by Philipps-University Marburg (Germany) and Nipah virus-Malaysia, NiV-M, (1.39 × 10^7^ PFU/mL) provided by the Rocky Mountain Laboratories (USA). Infectious work handling EBOV-GFP and NiV-M was performed in the Biosafety Level 4 (BSL-4) laboratory at the Robert Koch-Institute (Berlin, Germany).

### Cells and cell culture

Vero E6 (African green monkey kidney cells) were purchased from ECACC (Sigma Aldrich, #85020206) and cultured in Dulbecco’s Modified Eagle’s Medium (DMEM, Sigma-Aldrich, D6546) supplemented with 5% fetal bovine serum (FBS, PAN Biotech, P30-3306) and 1% Penicillin/Streptomycin/L-Glutamine (Fischer Scientific GmbH, 10,378,016). Cells were maintained at 37 °C in a humidified 5% CO_2_ atmosphere. Titer of EBOV-GFP and NiV-M were determined as Fifty-Percent Tissue Culture Infectious Dose (TCID_50_). For TCID_50_ assays, cells were seeded into 96-well plates (Faust Lab Science GmbH, TPP92696) at a density of 3 × 10^4^ cells/well and incubated at 37 °C and 5% CO_2_.

### Preparation of serum-cleared sheep blood

To mimic the presence of organic soiling under realistic contamination scenarios, we used serum-cleared sheep blood and bovine serum albumin (BSA) as representative proteinaceous matrices. These substances are commonly employed in disinfection and stability studies to simulate the protective effect of biological fluids on viral particles (19). For experiments carried out with sheep blood, a stock solution of bovine serum albumin (Roth, # 8076.4; Albumin Fraction V NZ-Origin) was prepared by dissolving 3 g BSA in sterile PBS in a total volume of 100 mL. To prepare the sheep blood, 8 mL of blood were centrifuged at 800 × g for 10 min. The supernatant was removed and erythrocytes were resuspended with PBS. The centrifugation and liquid removal steps were repeated until the supernatant remained clear. Thereafter, 1.5 mL blood were resuspended in 48.5 mL BSA solution.

### Stability assay

To test the influence of environmental conditions on the stability of virus particles, two scenarios were chosen regarding the combination of temperature (°C) and humidity (relative humidity, RH). First, conditions were selected which correspond to those in air-conditioned rooms (21 °C/40% RH) and, second, conditions found in, e.g., Germany during the warm summer months (28 °C/80% RH). To maintain a constant humidity and temperature, the samples were incubated in an environmental cabinet for the indicated period up to a maximum of 112 days (d). Stability of RG-4 pathogens was assessed on seven materials commonly found in BSL-4 laboratories as well as hospital isolation wards: glass, stainless steel, PVC/PET, nitrile, natural rubber, cotton fabric, and plastic (Table 1).

**Table 1:** Summary of selected materials used in the study including additional information.

Material	Additional information
PVC/PET	BSL-4 personal protection suit, Honeywell GmbH, Germany
Natural rubber	Ansell, Germany
Nitrile	Neo Touch, Ansell, Germany
Glass	D263 T eco, grain 800 = 7 μm, roughness = 0.3 Zell Quarzglas GmbH, Germany
Stainless steel	VA 1.4031 quality 2 B, t = 1.5 mm
Cotton fabric	Otto Wonneberger Nachf. GmbH, Germany
Plastic	TPP®, Trasadingen, Switzerland

From each individual material discs with a diameter of 10 mm were punched out. The direct usage of the plastic ground of 48-well plate dishes to mimic plastic surface is the only exception to this procedure. The distinct material discs were placed into a 48-well plate (TPP®; TPP92448). Considering the guidelines and procedures of the German Association for the Control of Viral Diseases (*Deutsche Vereinigung zur Bekämpfung der Viruskrankheiten e. V*., DVV) (19), two different testing solutions mimicking contamination were used: (1) DMEM/2% or (2) serum-cleared sheep blood in a 1:1 ratio.

Testing material was overlayed with 57.5 μL EBOV-GFP (2.2 × 10^5^ viral particles) or 10 μL (1.4 × 10^5^ viral particles) NiV-M viral stock diluted 1:1 in DMEM/2% or serum-cleared sheep blood. Mock treated samples were medium or blood only with constant volume. Samples were further incubated at 21 °C/40% RH or 28 °C/80% RH for indicated times. After that incubation period, the remaining viral particles were recovered from the test material by adding 185 μL (for EBOV-GFP samples) or 280 μL (for NiV-M samples) of 0.05 M Hepes buffer with a pH of 7.2 reaching a maximum volume of 300 μL. Subsequently, the mixture was incubated on a shaker (Thermo-Shaker PHMP-4; Grant-bio) at 800 rpm for 30 min at room temperature, ensuring thorough resuspension.

Following resuspension, 140 μL of the supernatant were employed in the TCID_50_ assay, while 135 μL were used for RNA extraction, subsequently followed by RT-qPCR. A proprietary control plasmid (5 μL) was added into each PCR sample as an internal standard to assess extraction efficacy (data not shown).

### Fifty-percent tissue culture infectious dose (TCID_50_) assay

Infectivity of viral particles was assessed for each solution on every material under the two selected environmental conditions described above by the tissue culture infectious dose (TCID_50_) assay. TCID_50_ refers to the concentration of a virus solution at which 50% of the cells are infected. Infection is assessed by qualitative determination of the cytopathic effect (CPE) induced by viral replication. TCID_50_ was determined as previously described (20) with minor changes. In brief, Vero E6 cells were grown overnight to 80–90% confluency in 96-well plates (Neolab, # 267334). Serially diluted virus samples in DMEM/2% (100 μL volume) were added onto the cells in technical triplicates, achieving a final volume of 200 μL per well. Viral titer was determined scoring for CPE (NiV-M) or GFP-positive cells (EBOV-GFP) after a minimum of 7 days and TCID_50_/mL was calculated using the Spearman & Kärber algorithm (20).

### Viral passaging

**Figure 1:**
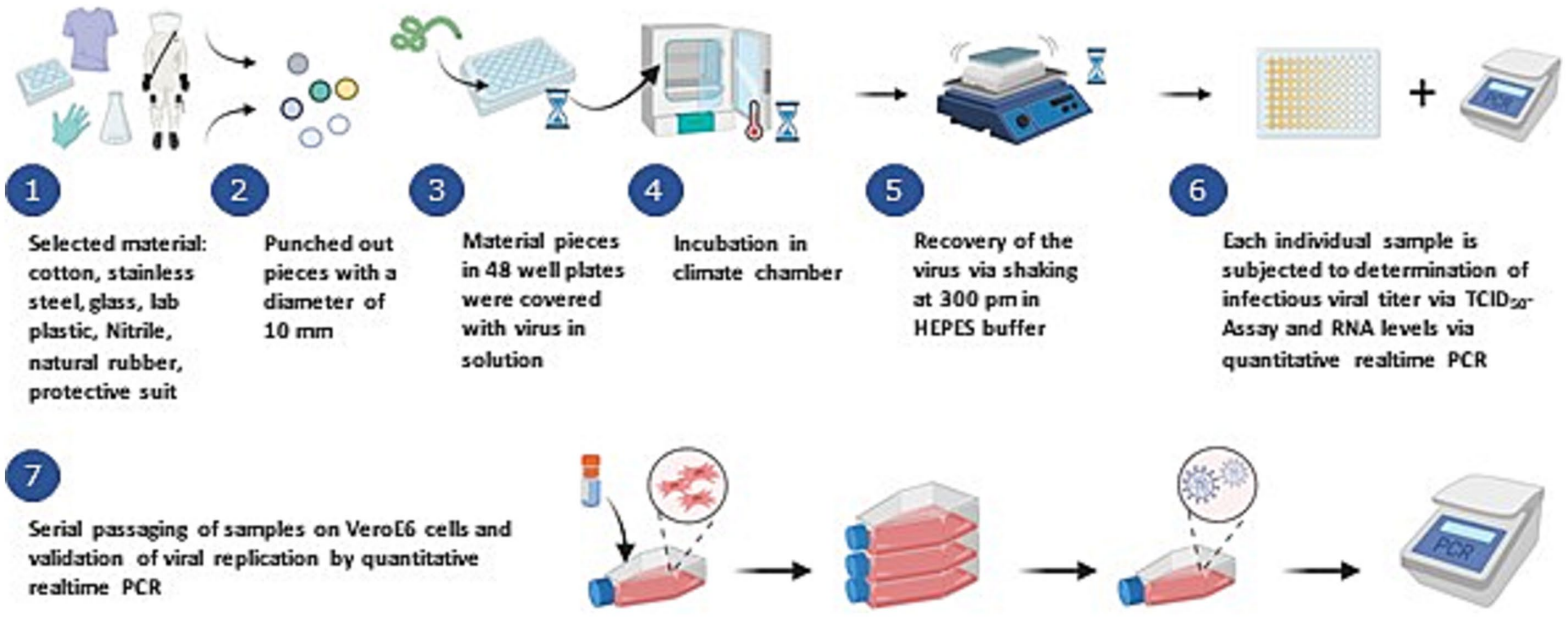
Schematic depiction of the complete workflow of the testing approach. Steps 1–6 represent the general approach to evaluate the viral particle stability and infection retention on the indicated testing material under two distinct environmental conditions. Step 7 further describes the validation of the stability analyses results using serial passaging on VeroE6 cells to facilitate viral propagation of any particle which might still be infectious as a second analysis checkpoint. Created with Biorender.com.

Given the limited sensitivity of the TCID₅₀ assay, serial passaging on VeroE6 cells was performed, as shown in Figure 1, to detect any residual infectious particles. These samples—anticipated to be negative—served as a confirmatory evaluation of the TCID₅₀ results. Supernatants were collected at days 0 and 14 and analyzed by RT-qPCR. Non-infected controls were included as reference; they consistently showed no detectable Ct value or a Ct ≥ 38, which was attributed to technical background of the qPCR instrument. Any reduction in Ct value, irrespective of magnitude, was interpreted as evidence of viral gene expression. Active replication, and thus infectious virus production, was inferred when the day 14 Ct value was equal to or lower than that observed on day 0.

To this end, virus samples were added and recovered from the respective material at indicated times as described above. Subsequently, VeroE6 cells (10^5^/T25) were infected with the total test virus sample (maximal volume of 300 μL) in 10 mL DMEM/2% and incubation was continued for 1 week. Afterwards the supernatant (approx. 10 mL) was transferred onto naïve VeroE6 cells (10^5^/T25) and 2 mL fresh DMEM/2% was added to each culture flask. After an additional incubation for 1 week a sample of the supernatant subjected for RT-qPCR was taken and processed as described above. All incubation steps were carried out at 37 °C and 5% CO_2_ using a humidified incubator.

### RNA extraction and RT–qPCR

Supernatant recovered from the testing materials containing the remaining liquid and resuspended with 0.05 M Hepes buffer or directly taken from culture flasks depending on the analysis approach was collected in AVL-buffer (QIAGEN) and mixed with an equal volume of 100% ethanol. RNA extraction was carried out using the QIAamp Viral RNA Mini extraction kit (QIAGEN, # 52906) according to the manufacturer’s instructions. RNA was eluted into 50 μL nuclease-free H_2_O. Quantitative RT–PCR for EBOV detection was performed using the AgPath-ID™ One-Step RT–PCR Kit (Life Technology, # 4387391 M) with 3 μL RNA input.

The following primers were used for EBOV-GFP (10 μM final concentration each): forward primer 5’ACTCCTACTAATCGCCCGTAAG-3′, reverse primer 5′- ATCAGCCGTTGGATTTGCT-3′, detection probe 5’ FAM-CACCCAAGGACTCGC-BBQ-3′ (TIB Mol Bio). Thermocycling conditions: 15 min/45 °C, 10 min/95 °C and 45 amplification cycles consisting of 15 s/95 °C and 45 s/60 °C. Quantitative RT–PCR for NiV-M detection was performed using the AgPath-ID™ One-Step RT–PCR Kit (Life Technology, # 4387391 M) with 5 μL RNA input. Following primers were used for NiV-M: forward primer 5′- GTTCAGGCTAGAGAGGCAAAATTT −3′, reverse primer 5′- CCCCTTCATCGATATCTTGATCA −3′, detection probe 5’ FAM- CTGCAGGAGGTGTGCTCATTGGAGG -BBQ -3′ (TIB Mol Bio). Thermocycling conditions: 15 min/45 °C, 10 min/95 °C and 45 amplification cycles consisting of 15 s/95 °C and 60 s/60 °C.

Reactions were performed on a C1000 Touch BioRad cycler and analyzed using CFX96 CFX Maestro Version 4.1.2433.1219. Ct-value conversion to genomic copy numbers/mL were calculated by qPCR of serially diluted extracts of a quantified infectious *in vitro* transcript of EBOV-GFP or NiV-M stock (concentrations ranging from 10^2^ to 10^6^ copies/μL).

### Data analysis

Data analysis was performed using Graph-Pad Prism 9.1.0 (221) software (Inc., La Jolla, CA, USA).

## Results

A schematic overview of the complete workflow is depicted in Figure 1. Infectious viral titer of indicated samples were determined at day 1, 2, 7, 14, 28, 56, and 112. Accordingly, we can only extrapolate the infectivity of samples that fall between two analysis time points (Table 2).

**Table 2:** Summary of maximum retention of infectivity of EBOV-GFP and NiV-M.

Condition	21 °C, 40% RH		28 °C, 80% RH	
	Blood	Medium	Blood	Medium
EBOV-GFP				
PVC/PET	28	28 (2)	14	14 (1)
Natural rubber	28	28	28	14 (1)
Nitrile	56	28	28	2
Glass	28	28	112	14 (7)
Stainless steel	28	28	28	7
Plastic	28	28	56	28
Cotton fabric	28	14	28	14
NiV-M				
PVC/PET	28 (7)	14 (2)	14 (2)	7
Natural rubber	28 (7)	14 (2)	28 (2)	14
Nitrile	14	2 (1)	7	2
Glass	28 (14)	14	28	28
Stainless steel	14	14 (7)	28 (2)	28
Plastic	28 (14)	14	14	112 (28)
Cotton fabric	7	14 (2)	7	7

**Figure 2:**
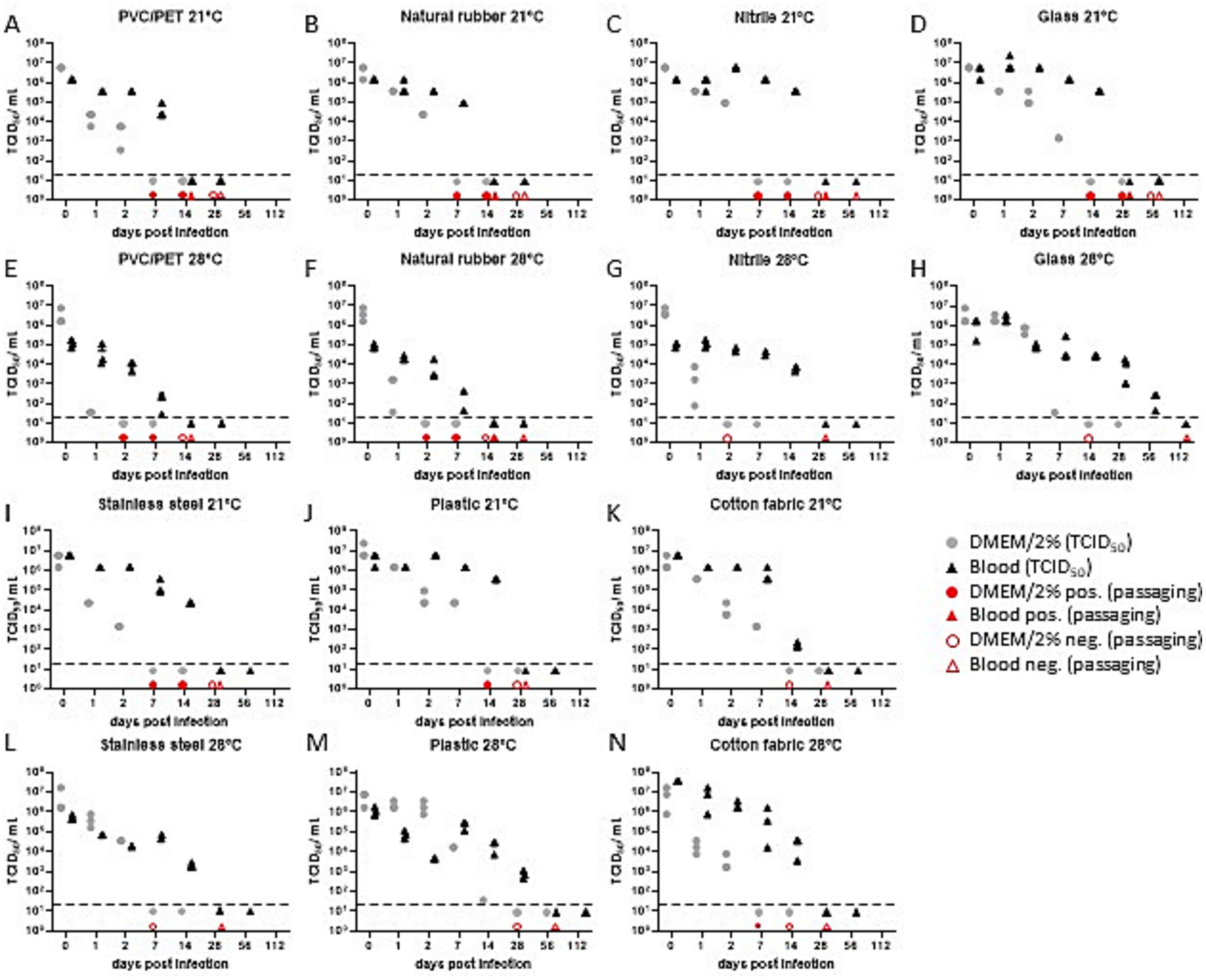
Virus titer determined by the TCID_50_ assay for EBOV-GFP under indicated conditions **(A–N)**. Gray and black filled symbols represent the individual samples taken in triplicates at each time point. TCID_50_ assay sensitivity cut-off is marked as dotted line. Sample points below were tested negative in the TCID_50_ assay. Red symbols indicate data points which showed either active viral replication (filled) or no viral replication (empty) (DMEM/2% = DMEM supplemented with 2% FCS, blood = serum-cleared sheep blood).

**Figure 3:**
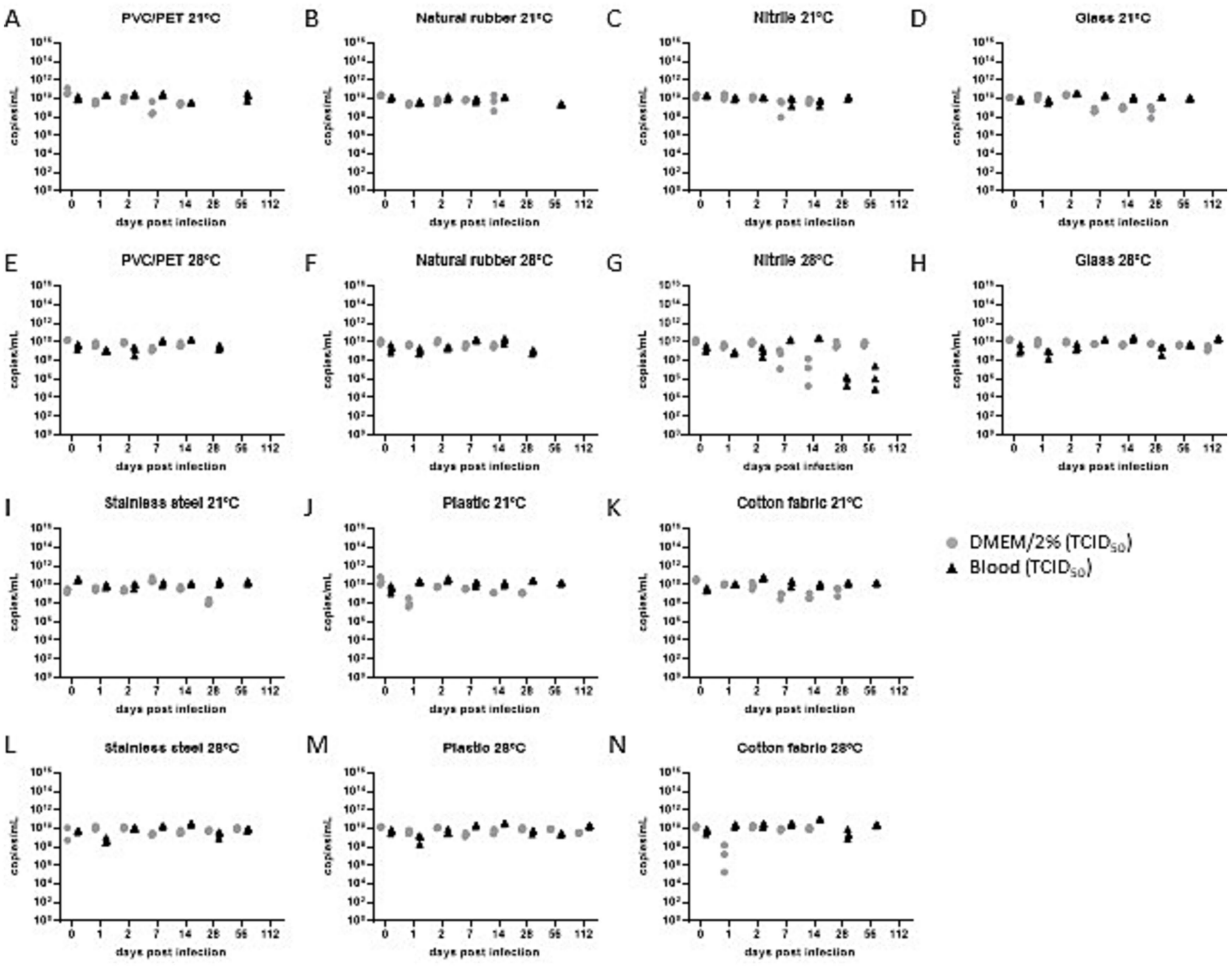
Virus titer determined by the TCID_50_ assay for NiV-M under indicated conditions **(A–N)**. Gray and black filled symbols represent the individual samples taken in triplicates at each time point. TCID_50_ assay sensitivity cut-off is marked as dotted line. Sample points below were tested negative in the TCID_50_ assay. Red symbols indicate data points which showed either active viral replication (filled) or no viral replication (empty) (DMEM/2% = DMEM supplemented with 2% FCS, blood = serum-cleared sheep blood).

Listed are the testing time points (in days), at which no infectious particles were detected in indicated samples based on the data shown in Figures 2, 3. The time point at which a 4-log reduction in viral titer was determined using the TCID_50_ assay is indicated in parentheses, if it differs from the time point at which no viable particles were detected.

In all mock-infected samples, neither infectious virus particles nor viral RNA were detected by the applied TCID_50_ assay or RT-qPCR, respectively. Reliability of the testing approach was confirmed by RT-qPCR specific to EBOV and NiV-M. Viral RNA was detected at all analysis timepoints throughout the study (day 0–112) with comparable levels of viral genome equivalents (Supplementary Figures S1, S2).

### No detection of infectious EBOV particles at day 112

We found EBOV-GFP particles being equally or less stable in DMEM/2% compared to samples supplemented with serum-cleared sheep blood regardless of the testing material or applied condition (Figure 2; Table 2; Supplementary Table S1). When applying condition 1 (21 °C/40% RH), no infectious viral particles were found during validation throughout the passage assay in samples incubated in DMEM2% on day 14 on glass, or day 28 for all other materials, while samples supplemented with serum-cleared sheep blood were tested as non-infectious after 28 days or later. Nitrile represented an exception in the analysis, with no infectious particles detected on day 56 (Figures 2A–D, I–K). A change in the incubation conditions to 28 °C/80% RH resulted in equal (plastic) or lower retention of infectious viral particles (cotton fabric, stainless steel, nitrile, natural rubber) when incubated in DMEM/2% (Figures 2E–H, L–N; Table 2; Supplementary Table S1). The addition of serum-cleared sheep blood led to increased retention times (PVC/PET) or similar retention times (cotton fabric, stainless steel, nitrile, natural rubber). Exceptions were detected for plastic (56 days) or glass (112 days), both representing very smooth surfaces compared to the rest of analyzed materials (Figures 2E–H, L–N; Table 2; Supplementary Table S1). In addition, the decay of infectious virus particles was gradual when incubated in DMEM/2%, while the stability of particles incubated with serum-free sheep blood was more lagging.

The stability assessment of NiV-M particles revealed a moderate retention of infectivity, with minimal influence observed from the introduction of blood or alterations in climate parameters.

Notably, the overall stability of NiV-M particles was comparable, or lower than that of EBOV-GFP across all examined conditions (Figure 3; Table 2; Supplementary Table S2). Intriguingly, the most prolonged retention of infectious particles was observed in samples diluted in DMEM/2% and incubated on plastic at 28 °C/80% RH (Figure 3M; Supplementary Table S1), followed by incubation on various surfaces including PVC/PET (21 °C/40% RH, blood), natural rubber (21 °C/40% RH and 28 °C/80% RH, blood), glass (21 °C/40% RH and 28 °C/80% RH, blood), plastic (21 °C/40% RH, blood, and 28 °C/80% RH, DMEM/2%), and stainless steel (28 °C/80% RH, blood, and DMEM/2%). Across all other samples, the latest timepoint at which no infectious viral particles were detected ranged from 2 to 28 days, irrespective of the substrate upon which they were incubated. Notably, the majority of samples exhibited a gradual loss of infectivity over time.

Of particular interest, the addition of blood or introducing changes in environmental conditions, including elevated temperature and increased humidity, exerted only minor effects on the stability of the virus particles. The present data are influenced by environmental factors and additional parameters, such as the properties of the materials, preventing the derivation of predictive conclusions. Consequently, no general inference can be made regarding an extension of tenacity.

Our investigation of the infectivity of viral particles uncovered a gradual reliance on surface characteristics, as well as temperature and humidity variables (Table 2). Within the specified temperature and relative humidity parameters, our results show an absence of detectable infectious EBOV or NiV-M virus particles on the examined material between day 2 and day 112 depending on surface material and environmental condition. As determined by our analyzed timeline the median retention time ranges between 14 and 28 days. This underscores the intricate interplay between environmental conditions and viral persistence, highlighting the importance of understanding these factors in the context of viral transmission and control.

## Conclusion and discussion

This study systematically quantified the stability and retention of infectivity of two risk group 4 viruses—*Orthoebolavirus* and *Henipavirus*—under defined environmental conditions, generating detailed experimental data across multiple materials relevant to laboratory and patient care settings. Controlled scenarios simulated contamination from infected body fluids (patient management) or viral suspensions (laboratory spills), thereby providing a robust evidence base for understanding virus survival dynamics outside the host.

The results show that both viruses can remain infectious for several days after deposition on various surfaces. Persistence was strongly influenced by the suspension medium (DMEM/2% FBS or serum-cleared sheep blood) and environmental temperature. Additional environmental parameters not assessed in this study, including ultraviolet (UV) radiation and sample pH, are likely to influence viral infectivity. Consequently, the retention times reported here should be interpreted as upper-limit estimates under the tested conditions. While infectivity declined over time, the rate and duration of decline varied by virus type and condition: For *Orthoebolavirus*, blood-supplemented samples demonstrated increased stability, highlighting the prolonged hazard potential of blood-contaminated materials. *Henipavirus* generally exhibited shorter retention times, in line with previous findings (12, 15), though in some cases infectious particles persisted for up to 14 days.

Comparison with published work revealed both concordance and divergence. Piercy et al. (17) reported EBOV infectivity in liquid media for up to 46 days at 4 °C, consistent with our observation that lower temperatures prolong stability, even though incubation parameters differed. Similarly, Fisher et al. (18) demonstrated a strong temperature-stability correlation, with tropical conditions (28 °C, 80% RH) limiting *Orthoebolavirus* survival to 6 days, a trend also evident in our data. In contrast, very long survival times calculated for Zaire ebolavirusΔVP30 in blood droplets within syringe needles (189.9 days at 21 °C, 55% RH) (21) could not be reproduced in our experiments, emphasizing the importance of empirical verification for extrapolated persistence estimates.

Within our study design, the texture of the tested material did not exert a consistent overall effect on viral stability. However, certain virus–condition combinations showed tendencies—such as reduced NiV-M stability on cotton fabric, natural rubber, and PVC/PET at 28 °C/80% RH—that were not mirrored for EBOV-GFP. Literature synthesis by Vasickova et al. (22) suggests that non-porous surfaces generally support longer viral viability than porous ones, with some exceptions. In our experiments, *Orthoebolavirus* remained viable longest on glass, whereas *Henipavirus* showed maximal persistence on plastic.

RT-qPCR analyses detected viral RNA for up to 112 days without significant decrease in genome copy number under all tested conditions (Supplementary Figures S1, S2). This decoupling of RNA detection from infectivity underscores that PCR-based surface assays cannot serve as reliable proxies for infectious risk assessment.

By quantifying time-dependent infectivity loss under “dirty conditions” (23), our results provide defined intervals at which infectious viral loads fall by ≥ 4 log levels—comparable to the effectiveness thresholds for chemical disinfection set by the German Association for Combating Viral Diseases (DVV) and the Robert Koch-Institute (19) —and, under certain conditions, meet inactivation criteria of EN 12740:1999 (e.g., via autoclaving). Serial passage on VeroE6 cells confirmed the absence of residual infectivity beyond these experimentally defined survival limits.

By mimicking conditions in which contamination of surfaces occurs via body fluids of infected patients, the workflow of our study limits the uncertainty at which time the virus containing liquid was actually dried onto the respective material. Moreover, the porous nature of the material, as seen on cotton fabric, represents a crucial factor affecting demonstrated results.

The main route of person-to-person transmission of *Orthoebolavirus* and *Henipavirus* is the contact with contaminated body fluids harboring high viral load. Therefore, family members of infected individuals, as well as health care workers, are at the highest risk of becoming infected compared to the rest of the population (9, 10). The lack of systematic experimental, data-based knowledge about the persistence of highly pathogenic viruses under distinct environmental conditions causes uncertainties regarding patient management (e.g., quarantine or isolation), and the selection of personal protective clothing of patient caretakers.

Our findings provide a robust scientific basis for understanding the environmental stability of high-risk viral agents under defined conditions, thereby directly informing evidence-based risk assessments in both clinical and laboratory settings. While the study does not directly evaluate the efficacy of personal protective equipment (PPE), insights into viral persistence on surfaces can support biosafety planning, optimize decontamination strategies, guide PPE policies, and reinforce the importance of timely protective measures. The primary contribution of this work lies in providing condition-specific infectivity data that enable more precise patient management and laboratory decision-making than reliance on precautionary assumptions alone.

## Data Availability

The original contributions presented in the study are included in the article/Supplementary material, further inquiries can be directed to the corresponding author.

## Supplementary material

SUPPLEMENTARY FIGURE S1 Viral RNA is recovered from the test material through the entire measuring time range (0-112 days) independently from the incubation conditions. Shown are the calculated genome equivalents per initial viral stock volume (copies per mL) placed onto the respective material at indicated conditions based on quantitative real-time PCR data specific to EBOV-GFP **(A–N)**. Symbols represent the individual samples taken in triplicates at each time point (DMEM/2% = DMEM supplemented with 2% FCS, blood = serum-cleared sheep blood).

SUPPLEMENTARY FIGURE S2 Viral RNA is recovered from the test material through the entire measuring time range (0-112 days) independently from the incubation conditions. Shown are the calculated genome equivalents per initial viral stock volume (copies per mL) placed onto the respective material at indicated conditions based on quantitative real-time PCR data specific to NiV-M **(A–N)**. Symbols represent the individual samples taken in triplicates at each time point (DMEM/2% = DMEM supplemented with 2% FCS, blood = serum-cleared sheep blood).
