# Supplementary material for: Improving biosafety measures in high containment laboratories and patient care: a systematic analysis of Orthoebolavirus and Henipavirus stability
Source: Front Public Health. 2025 Nov 3;13:1648115. doi: 10.3389/fpubh.2025.1648115 (PMC12620354; doi:10.3389/fpubh.2025.1648115)
Supplement: Supplementary file 1 [file Data_Sheet_1.pdf]

Table S1: Individual results of *virus titer determined by the TCID$_{50}$ assay for EBOV-GFP virus after indicated climate conditions.*

days	21°C medium			21°C blood			28°C medium			28°C blood		
**PVC/PET**												
0	5885654,4	5912902,8	5885654,4	1487762,64	1487762,64	1479588,12	7673400	1649520	1649520	71221,1923	113056,596	179466,159
1	23460,8724	5803,9092	23024,898	373303,08	370578,24	373303,08	35,5482	35,5482		17889,9546	11323,5491	113056,596
2	5803,9092	362,40372	5749,4124	373303,08	370578,24	373303,08				11323,5491	11323,5491	4493,75343
7				93462,012	23024,898	23024,898				27,6207783	278,4	
**Natural rubber**												
0	5912902,8	1487762,64	5885654,4	1487762,64	1487762,64	1487762,64	3554820	7673400	1649520	113056,596	71221,1923	71221,1923
1	373303,08	370578,24	373303,08	1479588,12	373303,08	373303,08	1649,52	35,5482	1649,52	28398,5328	17889,9546	17975,0137
2	23460,8724	23024,898	23460,8724	373303,08	373303,08	370578,24				17975,0137	2779,60169	2779,60169
7				93462,012	93462,012	93734,496				441,932453	43,8452525	
**Nitril**												
0	5912902,8	5912902,8	5912902,8	1487762,64	1487762,64	1487762,64	3554820	7673400	3554820	113056,596	71221,1923	113056,596
1	373303,08	373303,08	373303,08	1479588,12	373303,08	1479588,12	1649,52	76,734	7673,4	113056,596	71559,8186	179466,159
2	93734,496	93462,012	93734,496	5885654,4	5885654,4	5912902,8				71221,1923	71221,1923	45079,8609
7				1487762,64	1479588,12	1479588,12				45079,8609	28398,5328	45079,8609
14				373303,08	373303,08	370578,24				7133,38893	4493,75343	7133,38893
**Glass**												
0	5885654,4	5885654,4	5885654,4	5885654,4	1487762,64	5885654,4	7673400	1649520	1649520	1649520	1649520	164952
1	373303,08	370578,24	370578,24	2,40058404e+007	5912902,8	5912902,8	3554820	1649520	1649520	1649520	3554820	1649520
2	373303,08	93734,496	93462,012	5912902,8	5885654,4	5912902,8	767340	767340	355482	71559,8186	113056,596	71221,1923
7	1452,33972	1452,33972	1452,33972	1479588,12	1487762,64	1487762,64	35,5482			28398,5328	283536,673	28398,5328
14				370578,24	373303,08	373303,08				28398,5328	28398,5328	28398,5328
28										11323,5491	1113,6	17889,9546
56										278,4	278,4	43,8452525
Metall												
0	1487762,64	5885654,4	5912902,8	5885654,4	5885654,4	5885654,4	1649520	1,64952e+007	1649520	450086,414	450086,414	714467,646
1	23024,898	23024,898	23460,8724	1487762,64	1487762,64	1487762,64	355482	767340	164952	71221,1923	71559,8186	71221,1923
2	1452,33972	1452,33972	1452,33972	1487762,64	1487762,64	1487762,64	35548,2	35548,2	35548,2	17889,9546	17889,9546	17889,9546
7				93734,496	93734,496	373303,08				71559,8186	45079,8609	71559,8186
14				23024,898	23024,898	23024,898				1751,03934	2779,60169	1751,03934
Plastic												
0	5885654,4	2,40058404e+007	5912902,8	1487762,64	5885654,4	5885654,4	1649520	7673400	7673400	1791826,29	1128779,83	714467,646
1	1487762,64	1479588,12	1479588,12	1487762,64	1487762,64	1487762,64	3554820	1649520	1649520	71221,1923	113056,596	45079,8609
2	93734,496	23024,898	23024,898	5885654,4	5912902,8	5885654,4	3554820	767340	1649520	4493,75343	4493,75343	4493,75343
7	23024,898	23460,8724	23460,8724	1487762,64	1487762,64	1487762,64	16495,2	16495,2	16495,2	113056,596	284884,769	283536,673
14				370578,24	373303,08	373303,08		35,5482		28398,5328	7133,38893	28398,5328
28										701,524041	1103,08566	441,932453
Cotton Fabric												
0	1487762,64	1487762,64	5885654,4	5912902,8	5912902,8	5912902,8	767340	7673400	1,64952e+007	3,55482e+007	3,55482e+007	3,55482e+007
1	370578,24	370578,24	370578,24	1487762,64	1487762,64	1487762,64	35548,2	7673,4	16495,2	7673400	1,64952e+007	767340
2	23460,8724	5803,9092	5803,9092	1487762,64	1487762,64	1479588,12	1649,52	1649,52	7673,4	3554820	1649520	1649520
7	1452,33972	1452,33972	1452,33972	1479588,12	373303,08	373303,08				16495,2	1649520	355482
14				144,144036	228,88656	144,144036				3554,82	35548,2	35548,2

Table S2: Individual results of *virus titer determined by the TCID$_{50}$ assay for Nipah virus after indicated climate conditions.*

days	21°C medium			21°C blood			28°C medium			28°C blood		
**PVC/PET**												
0	7,44e+007	7,44e+007	7,44e+007	7,44e+007	7,44e+007	7,44e+007	744000	744000	744000	7440000	7,44e+007	7,44e+007
1	7,44e+007	7440000	6690000	74400	7440000	7440000	7380	7380	7380	7440	7380	74400
2	738			74400	74400	74400	16050	7440	74400			244,5
7				113,4	738							
14				244,5	113,4							
**Natural rubber**												
0	7,44e+008	7,44e+007	7,44e+007	7,44e+007	7,44e+007	7,44e+007	74400	744000	74400	7440000	7,44e+007	7,44e+007
1	160500	744000	74400	7440000	7440000	7440000	7380	7440	528	7380	7440	7440
2			528	74400	744000	744000	244,5	113,4	113,4	113,4	244,5	113,4
7		113,4	244,5	528								
**Nitril**												
0	7,44e+008	7,44e+007	7,44e+007	7440000	7,44e+007	7440000	74400	7380	74400	7,44e+007	7440000	7440000
1	7380	7380	738	7440000	7440000	7440000	738	7380	528	744000	744000	74400
2				744000	7440000	7440000				744000	744000	74400
7				74400	74400	74400						
**Glass**												
0	7440000	7440000	7440000	7440000	7,44e+007	7440000	74400	744000	74400	744000	744000	744000
1	7440000	7,44e+007	7440000	7440000	7440000	7440000	74400	744000	74400	74400	744000	744000
2	74400	74400	7380	7440000	7440000	7440000	74400	74400	74400	74400	7380	74400
7	0	0	0	74400	744000	744000	74400	74400	7380			
**Metall**												
0	7440000	7,44e+007	7440000	7,44e+007	7,44e+007	7,44e+007	7440000	744000	74400	7,44e+007	7,44e+007	7,44e+008
1	7,44e+007	7,44e+007	7,44e+007	744000	744000	7440000	74400	74400	74400	7,44e+007	7,44e+007	7,44e+007
2	7,44e+007	7,44e+007	7,44e+007	744000	744000	744000	74400	74400	74400	7440	7380	7380
7	244,5	244,5	244,5				16050	74400	16050			
**Plastic**												
0	7,44e+007	7,44e+007	7,44e+007	7,44e+007	7,44e+007	7,44e+007	744000	744000	160500	7,44e+007	7,44e+007	7440000
1	7,44e+007	7,44e+007	7,44e+007	74400	744000	744000	744000	744000	744000	7,44e+007	7,44e+007	7,44e+007
2	744000	744000	744000	744000	744000	744000	160500	744000	744000	744000	744000	744000
7	7380	7440	7380	74400	7380	74400	744000	744000	74400			
14				244,5	113,4		113,4	244,5	113,4			
**Cotton Fabric**												
0	7,44e+007	7440000	7,44e+007	7440000	7440000	7,44e+007	7440	74400	1590	744000	744000	744000
1	7440	7440	7440	7440000	7440000	7440000	7380	738	738	738	738	528
2	113,4	528	738	74400	744000	7440000	244,5	113,4	738			
7		738	528									
