# Supplementary figures and images for: Improving biosafety measures in high containment laboratories and patient care: a systematic analysis of Orthoebolavirus and Henipavirus stability
Source: Front Public Health. 2025 Nov 3;13:1648115. doi: 10.3389/fpubh.2025.1648115 (PMC12620354; doi:10.3389/fpubh.2025.1648115)

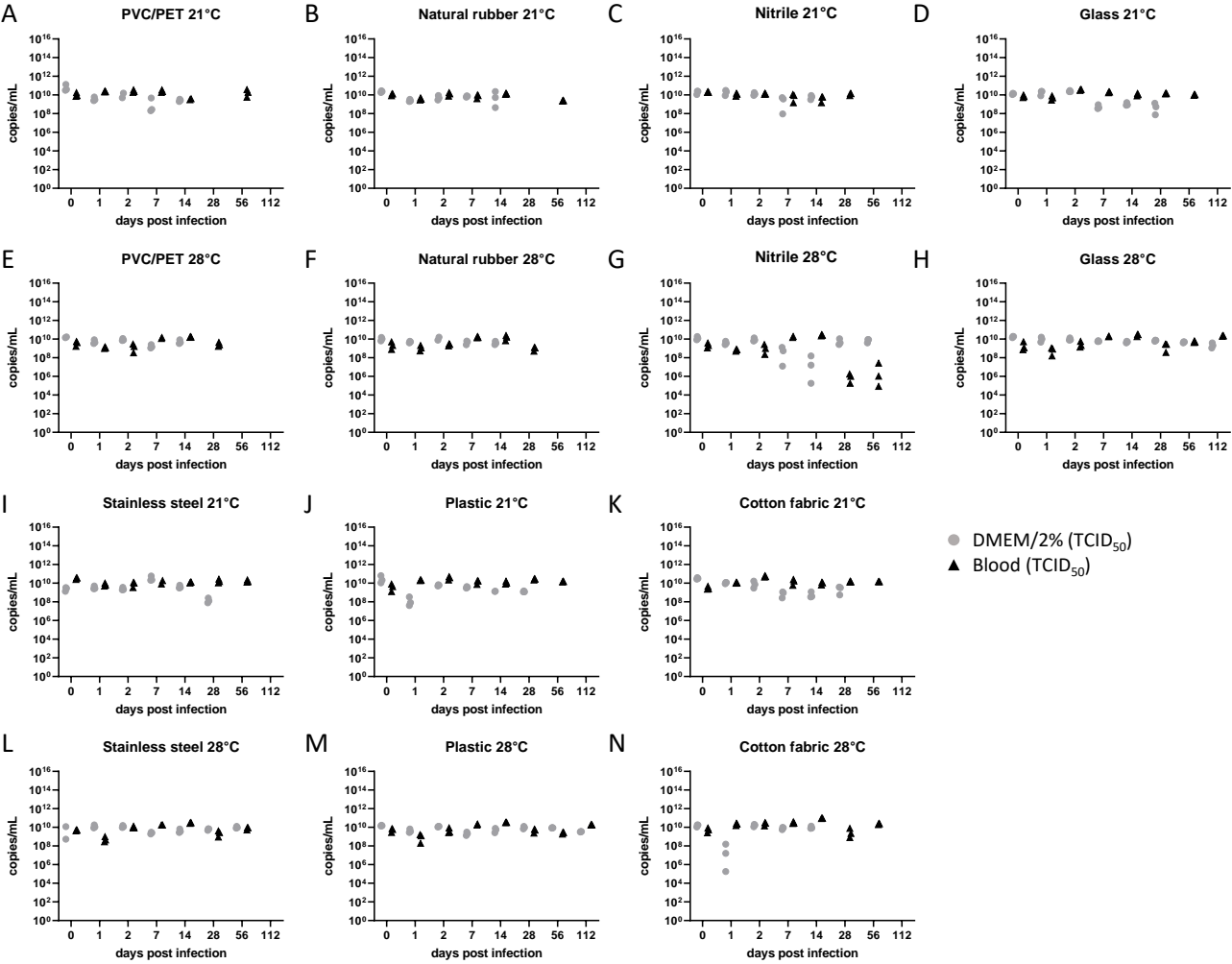

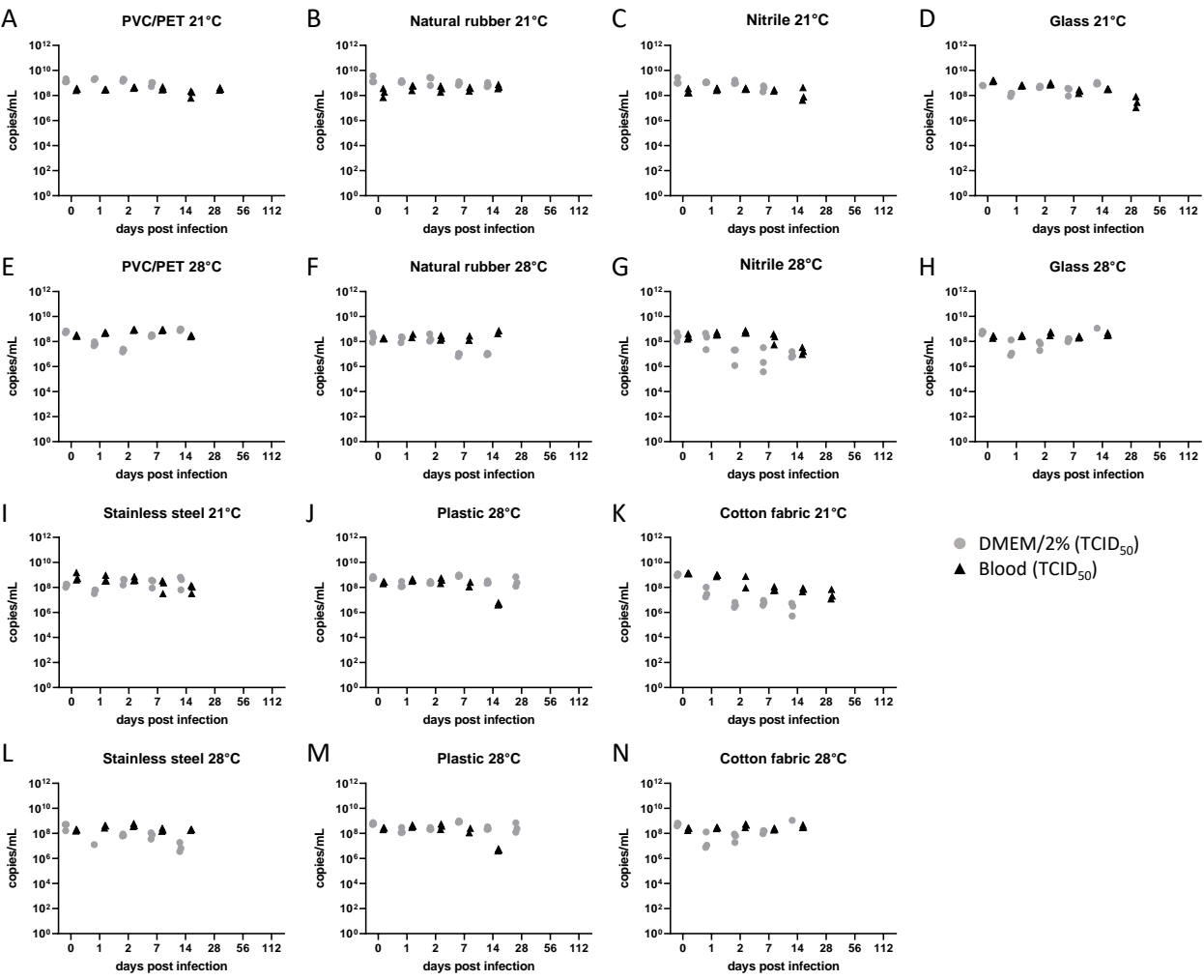

Supplement: Supplementary file 2 [file Supplementary_file_1.pdf]
